# Environmental processes and health implications potentially mediated by dust‐borne bacteria

**DOI:** 10.1111/1758-2229.13222

**Published:** 2023-12-27

**Authors:** Pengfei Hu, Yehonatan Sharaby, Ji‐Dong Gu, Adi Radian, Naama Lang‐Yona

**Affiliations:** ^1^ Civil and Environmental Engineering Technion—Israel Institute of Technology Haifa Israel; ^2^ Environmental Science and Engineering Research Group Guangdong Technion—Israel Institute of Technology Shantou Guangdong China; ^3^ Guangdong Provincial Key Laboratory of Materials and Technologies for Energy Conversion Guangdong Technion—Israel Institute of Technology Shantou Guangdong China; ^4^ Present address: Department of Biology and Environment University of Haifa Oranim Tivon Israel

## Abstract

Understanding microbial migration and survival mechanisms in dust events (DEs) can elucidate genetic and metabolic exchange between environments and help predict the atmospheric pathways of ecological and health‐related microbial stressors. Dust‐borne microbial communities have been previously characterized, but the impact and interactions between potentially active bacteria within transported communities remain limited. Here, we analysed samples collected during DEs in Israel, using amplicon sequencing of the 16S rRNA genes and transcripts. Different air trajectories and wind speeds were associated not only with the genomic microbial community composition variations but also with specific 16S rRNA bacterial transcripts. Potentially active dust‐borne bacteria exhibited positive interactions, including carbon and nitrogen cycling, biotransformation of heavy metals, degradation of organic compounds, biofilm formation, and the presence of pathogenic taxa. This study provides insights into the potential interactive relationships and survival strategies of microorganisms within the extreme dust environment.

## Graphical abstract

Investigating east Mediterranean dust events using gene and transcript amplicon sequencing reveals links between environmental factors, community composition, and bioactivity. Transcriptionally active species that exhibited positive interactions, included bacteria pivotal in carbon/nitrogen cycling, degradation, biofilm formation, and pathogenic potential. We provide insights into microbial activity and survival within the dust environment.

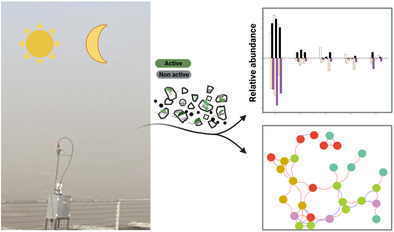

## INTRODUCTION

Airborne microorganisms disperse across continents and oceans, facilitating genomic and metabolic exchanges across different ecosystems (Tastassa et al., 2023). Moreover, they can significantly impact aquatic, atmospheric, and terrestrial environments and human health (Lang‐Yona et al., 2020; Maki et al., 2022; Risely et al., 2018; Sadakane et al., 2022). Extensive research has focused on dust‐borne microbial diversity including bacteria, eukaryotes, archaea and viruses (Aalismail et al., 2019), with the majority being the bacterial group, with predominance of Actinobacteriota, Bacteroidota, Firmicutes and Proteobacteria (Aalismail et al., 2019; Elmassry et al., 2020; Erkorkmaz et al., 2023; Gat et al., 2021). *Bacillus* species were detected in Asian dust events (DEs) and several East Asian countries (An et al., 2013; Maki et al., 2014), and are known for spore‐forming, which provides protection and enables long‐distance atmospheric transport (An et al., 2013; Griffin, 2004; Wainwright et al., 2003).

Dust storms facilitate extensive transport of microbiota across vast distances, subjecting them to harsh conditions (Goudie et al., 2006). Therefore, both viable and non‐viable microorganisms are present within the dust‐transported microbiota. Distinguishing between active and inactive microbes offers meaningful ecological insights into microbial survival strategies and their interactions with the dust environment (Schuerger et al., 2018). A few studies have utilized ribosomal RNA (rRNA) sequencing to characterize the potentially active airborne bacterial community (Blazewicz et al., 2013; Erkorkmaz et al., 2023; Šantl‐Temkiv et al., 2018), focusing primarily on the transcriptionally active microbial community structure and their relation to environmental factors such as air mass origination, atmospheric particulate matter (PM; <10 μm; PM10), and particle‐size (Erkorkmaz et al., 2023). However, the potential relation to meteorological parameters, diurnal changes, and the possible interaction mechanism and functionality within dust‐borne microbial communities remained elusive. Exploring these aspects could provide valuable insights into the survival strategies and transportation of potentially active microorganisms within the challenging dust macro‐environment. Furthermore, it may provide a deeper understanding of the impact of the dust microbiome on global biogeochemical cycles.

In this study we investigate bacterial communities carried over dust by utilizing high‐throughput amplicon sequencing of the rRNA gene (DNA) and transcript (RNA) to characterize these communities, assuming transcript represents potentially active communities, and construct a better understanding of their association to environmental parameters, and potential contribution to global cycles, ecosystems and health.

## RESULTS AND DISCUSSION

A series of three springtime DEs was sampled during 2022 on the rooftop of an eight‐story building, at the Technion Institute of Technology, Haifa, Israel (see Table 1). Day and night samples were collected in each event based on dust model prediction (www.meteoblue.com). Blank samples, of 30 s duration, were collected before each DE to ensure proper sampling and check for contaminations (see Supporting Information: Supporting Materials and Methods for more details). The bacterial community of the different DEs was characterized from the Metabarcode 16S‐rRNA gene sequencing dataset (see more details in Supporting Information: Supporting Materials and Methods, and Figure S1). Dust samples yielded at least 1893 high‐quality amplified sequence variants (ASVs) in 16S rRNA gene and 1667 in 16S rRNA (Tables S1 and S2, respectively).

**TABLE 1:** Sampling parameters, meteorological conditions and dust microbiome diversity parameters during sampled dust events (DEs).

Sample ID	Date	Sampling times	Sampling duration	Flow rate	Air volume	RH	Temperature	Wind speed	Wind direction	PM_10_	PM_2.5_	Richness			Diversity	
		UTC + 2	h	L min^−1^	m^3^	%	°C	m/s	°	μg	μg	Observe ASVs	Chao1	ACE	Shannon	Simpson
Blank I	29‐Mar‐22	11:40	0.008	14	0.007	26.15	22.22	4.63	122.87	0.14	0.07	‐	‐	‐	‐	‐
DE‐I_D_a	29‐Mar‐22	11:45–18:00	6.25	14	5.25	26	22.22	4.63	122.9	6898.50	3207.75	82	89.8	89.53	3.83	0.97
DE‐I_D_b												88	91.06	93.75	3.79	0.96
DE‐I_N_a	30‐Mar‐22	00:00–06:00	6	14	5.04	55	16.75	5.08	166.6	4573.80	2129.40	111	126.4	124.3	4.11	0.97
DE‐I_N_b												116	134	136.2	4.04	0.97
Blank II	06‐Apr‐22	10:55	0.008	14	0.007	61.41	20.63	7.78	244.08	1.50	0.39	‐	‐	‐	‐	‐
DE‐II_D_a	06‐Apr‐22	11:00–17:00	6	14	5.04	61	20.63	7.78	244.1	57,598.63	15,530.76	79	100.43	104	3.06	0.93
DE‐II_D_b												132	142.34	148.8	3.83	0.95
DE‐II_N_a	07‐Apr‐22	00:00–06:00	6	14	5.04	89	15.06	3.38	213.2	37,592.86	9974.66	22	22	22	2.46	0.89
DE‐II_N_b												43	43.25	43.69	2.86	0.9
Blank III	25‐Apr‐22	10:55	0.008	14	0.007	50.51	25.57	2.05	127.22	1.75	0.27	‐	‐	‐	‐	‐
DE‐III_D_a	25‐Apr‐22	11:00–17:00	6	14	5.04	51	25.57	2.05	127.2	154,714.90	17,604.72	125	136.81	145.2	3.18	0.88
DE‐III_D_b												168	201.44	197.2	4.02	0.95
DE‐III_N_a	26‐Apr‐22	00:00–06:00	6	14	5.04	46	18.42	1.26	231.8	35,4495.96	67,666.54	98	98	98.28	4.22	0.98
DE‐III_N_b												294	337.33	329.8	5.35	0.99

*Note*: DE samples and blanks, sampling rates and duration, diurnal state, meteorological parameters, as well as particle concentrations obtained from nearby stations, and richness and diversity parameters of the genomic data set obtained, are listed per the different samples.

Abbreviations: ACE, abundance‐based coverage estimator; ASV, amplified sequence variant; RH, relative humidity.

### Environmental variables impact on dust‐borne microbial community

To explore the potential influence of the meteorological and atmospheric factors on microbial survival and potential activity, we screened through the different parameters obtained from model calculations, and vicinal meteorological stations (see more details in Supporting Information: Supporting Materials and Methods). Substantial day/night fluctuations in relative humidity (RH) and temperature were observed during the DEs (Table 1). These factors are known to influence the viability of microbial communities in the air (Aarnink et al., 2015; Dannemiller et al., 2017; Haines et al., 2020). In addition, the lowest richness parameters were observed for DE‐II_N (Table 1), which might result from the elevated RH values detected during this sampled event, reaching 88.62% (Table 1). High RH levels might lead to an efficient deposition of PM (Kovbasyuk et al., 2022). In addition, previous studies indicate bacterial tolerance RH range of 40%–80% (Aarnink et al., 2015). Bacterial cells might not survive above this value due to an increase in osmotic pressure (Pepper et al., 2015).

Notably, DE‐II displayed significant day/night wind speed differences (7.78 m/s vs. 3.38 m/s, respectively). Wind speed has been previously linked with dust generation and bacteria transportation (Erkorkmaz et al., 2023; Fujiyoshi et al., 2017; Sorkheh et al., 2022; Ulrich, 2021), possibly associated with the observed microbial richness variation during this event, showing higher values for the daytime samples (see Table 1, Chao1 and abundance‐based coverage estimator [ACE] values).

The distinct origins of the three DEs were calculated by airmass backward trajectories (Figure S2). As can be seen, the sampled dust originated from different sources. South‐east origins, with air mass mainly originating from the mainland were observed for DE‐I, south‐west origin coming from Africa through the Mediterranean Sea, for DE‐II, and a mixed contribution of both east and northwest was observed for DE‐III. Interestingly, the daytime sample of this event was characterized mainly by east origins coming from inland Asia, while towards the end of sampling, origins shifted to originate from the Mediterranean Sea. The later was the main contribution of airmass in the nighttime sampling of this event.

**FIGURE 1:**
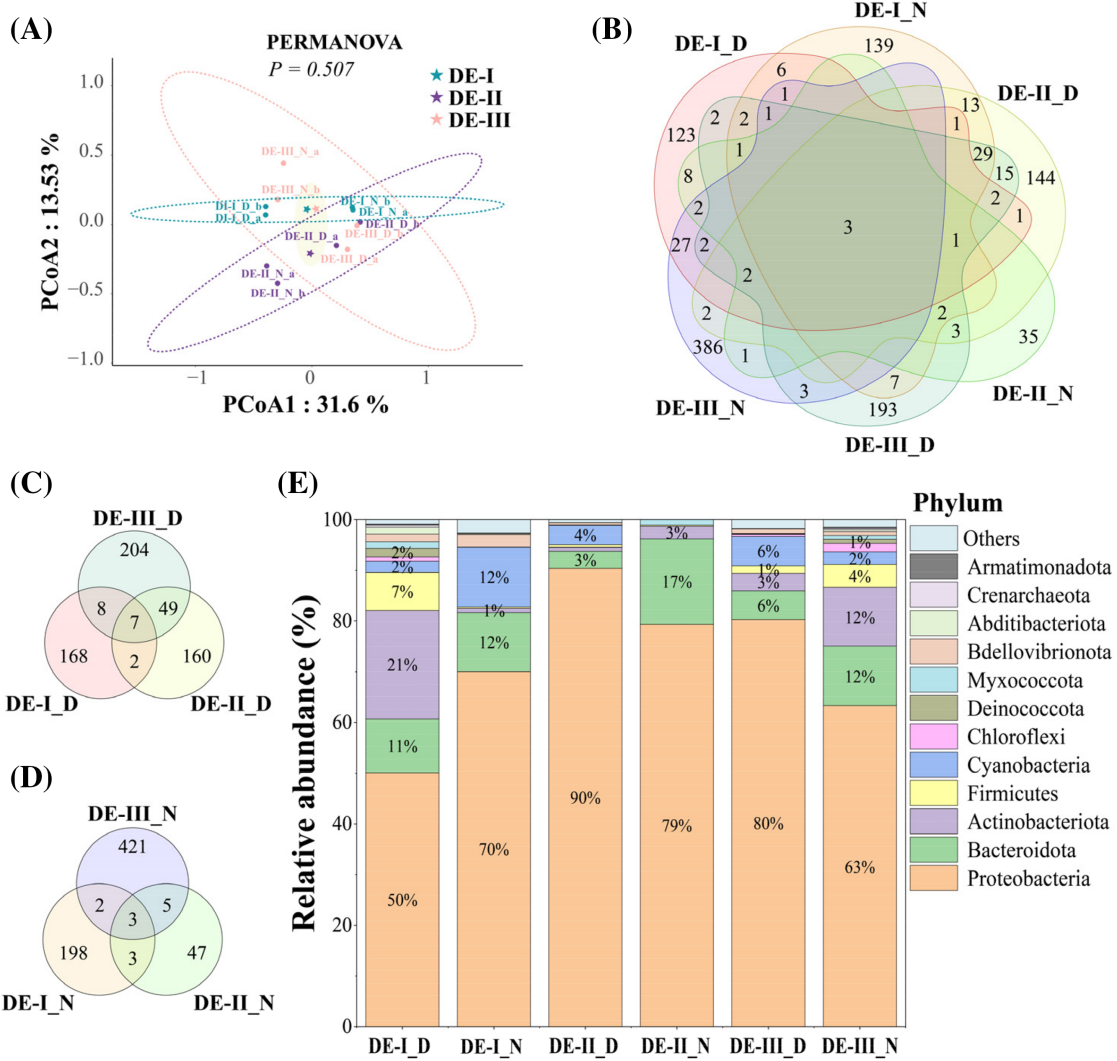
Bacterial community composition in dust samples collected during springtime in the East Mediterranean. (A) Principal coordinate analysis of the bacterial 16S rRNA gene and 16S rRNA ASVs, representing the genomic and transcriptional fingerprint of the dust microbiome, was conducted using Weighted UniFrac metrics. (B) Venn diagram illustrating the shared microbes among all sampled dust events. (C) and (D) diagrams present the shared microbes in the day and night samples, respectively. (E) The top 13 abundant phyla, ranked by average abundance, as represented in percentages for each dust sample. A detailed description of DNA/RNA extraction is given in the Supporting Information file. ASV, amplified sequence variant.

The particulate load has been linked with an increase in microbial richness (Erkorkmaz et al., 2023). A similar pattern was observed in our study, showing positive relations between higher PM concentrations (observed in DE_II and DE_III) and richness parameters (Chao1 and ACE values; Table 1). Previous studies have shown beta diversity clustering based on dust origins (Gat et al., 2017). In our study, such clusters can also be observed through PCoA (Figure 1A), nevertheless, less distinct, and with no significant differences through the PERMANOVA test. This might be due to the mixed origins of our samples, containing transport over marine and terrestrial environments, as well as diverse airmass sources (Figure S2).

### Dominant dust‐borne bacteria associated with environmental parameters

The Venn diagram revealed three ASVs shared among the six dust groups (Figure 1B), including Chitinophagaceae (ASV67), its specific genus, *Sediminibacterium* sp. (ASV589) and *Acidibacter* (ASV1248; Table S1). Both genera are ubiquitous in soils (Ling et al., 2022; Wu et al., 2021).

Shared taxa between smaller groups were closely associated with the dust origin and diurnal cycle. For example, DE‐II and III daytime samples shared 49 ASVs (Figure 1C; Table S1), accounting for 20.21% and 10.23% of the microbial communities, respectively. This aligns with air mass back trajectories of these events (Figure S2), indicating a significant transport over the marine environment, likely leading to shared microorganisms, including those associated with the marine environment (e.g., *Prochlorococcus* sp.). In addition, microbial composition differences may stem from the additional east origins of DE‐III air mass. In contrast, the nighttime samples exhibited relatively low rates of shared ASVs, with only three identified (Figure 1D). In addition, paired night samples shared significantly fewer ASVs compared to the equivalent pair of day samples (Figure 1C,D).

Our results indicate diurnal shifts in biodiversity patterns of dust samples and are likely due to airmass exposure to the Mediterranean Sea environment, and the increase in RH during nighttime. There is relatively little research on diurnal shifts in bioaerosols. Saari et al. (2015) utilized fluorescent technology to monitor concentration variations between day and night (Saari et al., 2015), Hu et al. (2020) explored diurnal pathogens diversity in urban settings (Hu et al., 2020), and Gusareva et al. (2019) explored airborne community composition, presenting robust diurnal repetitive dynamics in tropical air ecosystem (Gusareva et al., 2019). To our knowledge, diurnal variations in microbial composition in DEs have not been explored to date, presenting an opportunity to enhance our understanding of microbial migration dynamics during dust transport.

### Predominant bacterial types in dust samples

The airborne bacterial populations analysed from the 16S rRNA gene were composed of several bacterial types (Table S1 and Figure 1E). The predominant ASVs identified in the dust samples belonged to the phyla Proteobacteria, Bacteroidota and Actinobacteriota. Cyanobacteria and Firmicutes, common in marine and terrestrial environments, were also detected in the dust samples with some variation. Specifically, Proteobacteria accounted for 90% and 80% in DE‐II and III daytime samples, reducing to 79% and 63% during daytime, respectively. In contrast, the trend was the opposite in DE‐I (Figure 1E). Predominant taxonomic groups within Proteobacteria included Comamonadaceae (13.10% in DE‐I_D and 28.55% in DE‐II_N), *Pseudoalteromonas* (22.66% in DE‐II_D), *Alteromonas* (9.07% in DE‐I_N, 31.93% in DE‐II_D and 15.50% in DE‐III_D) and *Sphingomonas* (7.97% in DE‐II_D, 14.12% in DE‐II_N, 20.18% in DE‐III_D and 3.54% in DE‐III_N; Table S4). These genera are common in marine environments (Romanenko et al., 2007; Yoon et al., 2003), supported by the Mediterranean Sea influence observed by back trajectory analysis (Figure S2). Bacteroidota was observed as the second dominant phylum in all three night‐sampling events (11.65% in DE‐I_N, 16.84% in DE‐II_N and 11.71% in DE‐III_N), in contrast to the lower values in daytime samples (3% in DE‐II_D and 6% in DE‐III_D). Actinobacteriota constituted 21.13% of the microbial composition in DE‐I_D samples, but less than 10% in other samples, except DE‐III_N (12%).

### Potentially active dust‐borne microbial community

**FIGURE 2:**
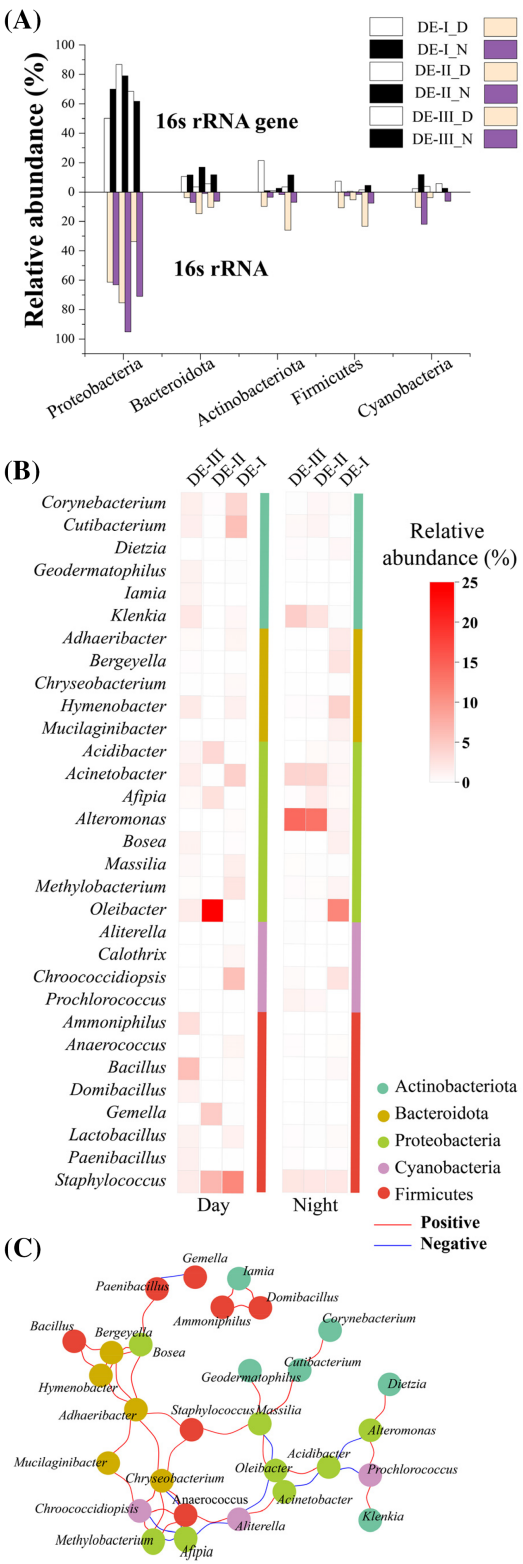
Relative abundance and interactions of bioactive dust‐borne bacteria. (A) A comparison of abundances of the top five phyla in dust events based on 16S rRNA gene (DNA) and 16S rRNA (cDNA) libraries, (B) a heatmap presenting the predominant potentially active genera within the top five phyla in day and night samples, and (C) a visualization of the interactions between different genera of the microbial community. The network analysis was constructed using Spearman correlation coefficients between genera. Nodes represent genera and edges represent correlations between nodes (Barberán et al., 2012; Varsadiya et al., 2021). A detailed description of the methodology is given in the Supporting Information file.

To characterize the potentially active microorganisms, we further analysed the sequenced 16S rRNA transcript, and compared it to the genomic 16S community (see Supporting Information: Supporting Materials and Methods for more details). Such a distinction between transcript indication for potentially active species and the genomic structure of the bacterial community may provide insights into the survival strategies of microbes and their interactions with the dust environment. In general, Proteobacteria dominated the 16S rRNA libraries, which reflects its likely abundance as an active phylum in the dust samples (Figure 2A). The abundance of other likely active microbial groups was less consistent compared to the 16S rRNA gene library. For example, DE‐I_D samples had a higher abundance of active Firmicutes, Cyanobacteria and Actinobacteriota than Bacteroidota. In the DE‐I_N night sample, bioactive Actinobacteriota and Firmicutes also exceed Bacteroidota, but there was an abrupt increase in the abundance of active Cyanobacteria. This may be attributed to the nighttime short air path through the Mediterranean Sea (Figure S2). In DE‐II, potentially active microorganisms were consistent with 16S rRNA gene relative abundance in general, apart from active Bacteroidota, which was less abundant in night samples of this DE, despite its higher abundance in the 16S rRNA gene results. In DE‐III daytime samples, the potentially active microorganisms were primarily clustered within Bacteroidota, Actinobacteriota and Firmicutes, with a relatively lower abundance of Proteobacteria. Specifically, Actinobacteriota and Firmicutes displayed relatively high activity, compared to a relatively low abundance of Actinobacteriota and Firmicutes in DE‐III_D 16S rRNA gene results (Figure 2A). In contrast, night samples showed no significant variation.

Genus‐level analysis shows diverse abundances of potentially active microorganisms within and between DEs (Figure 2B). In DE‐I daytime, *Staphylococcus*, *Chroococcidiopsis* and *Cutibacterium* exhibited relative abundances >5%, followed by *Acinetobacter*, *Corynebacterium,* and *Methylobacterium* with >2% relative abundance. DE‐I nighttime had highly active *Oleibacter*, *Hymenobacter*, *Chroococcidiopsis*, *Bergeyella,* and *Staphylococcus* (2%–5% relative abundance). Interestingly, in DE‐II, the potentially active *Oleibacter* also displayed high abundance during daytime (24.13%); however, this dynamic shifted during nighttime, where *Alteromonas* became the prevailing transcriptionally‐active microorganism (13.33%). In DE‐III, *Bacillus* emerges as the predominant active microorganism (6.02%), with night samples dominated again by *Alteromonas* (14.26%). These findings collectively suggest varying potentially active microbial compositions in different DEs.

The Mantel test conducted on potentially active bacterial communities from dust samples and environmental factors indicates positive correlations between all environmental variables (except for PM10) and bacterial taxa (Figure S3). This analysis highlighted significant associations between wind direction and four specific genera: *Corynebacterium* and *Cutibacterium*, from Actinobacteria, the proteobacterium *Massilia*, and *Lactobacillus*, from the Firmicutes phylum. These species seem to dominate our dust samples and might serve as key surviving species over dust.

### Survival strategies of potentially active dust‐borne microorganisms

To explore the co‐existence and biogeochemical contributions of the dust‐borne bacteria, we further analysed potential genes expressed in the bioactive community (see more details in Supporting Information: Supporting Materials and Methods). Adaptation strategies of microorganisms for surviving harsh conditions include mutual feeding and the formation of protective measures against environmental stressors (Haruta & Kanno, 2015; Thakur et al., 2022; Yin et al., 2019). Notably, such interactions appear to be prevalent within the core active microorganism communities of the sampled DEs (Figure 2C). The cycling of essential elements, such as carbon and nitrogen, plays a crucial role in the survival of microorganisms in unfavourable environments (Aasfar et al., 2021; Aronson et al., 2023; Bollmann et al., 2013; Jawaharraj et al., 2021; Sahoo et al., 2021; Yan et al., 2008). Our analysis revealed the presence of highly potentially active microorganisms engaged in carbon and nitrogen metabolism, suggesting a positive element cycle within the community. *Methylobacterium*, *Geodermatophilus*, *Bacillus* and *Dietzia* contribute to carbon availability through organic compound biodegradation (Kong et al., 2022; Sandhu et al., 2022; Venil et al., 2021; Yao et al., 2022; Yoshikawa et al., 2017; Zhang et al., 2013). *Pseudomonas* exhibited metabolic activity across all samples, suggesting active heterotrophic nitrification and aerobic denitrification processes over the dust particulates (Zhang et al., 2022). Additionally, the highly active *Corynebacterium*, *Mucilaginibacter* and *Acinetobacter* convert inorganic nitrogen compounds into ammonia and nitrate (Amrutha & Nampoothiri, 2022; Lee et al., 2016; Madhaiyan et al., 2010; Shelly et al., 2021), providing a stable nitrogen source for the microbial community. Previous studies have explored the contribution of dust to N_2_ fixation potentially from dust‐borne diazotrophs (Rahav et al., 2016; Rahav et al., 2018), but it remains to evaluate the direct contribution of the dust‐borne bacteria to this process.

### Metabolic potential of microorganisms carried over dust particles

High levels of dust particles can lead to adverse health effects associated with the presence of heavy metals, organic pollutant particles, and harmful minerals carried in the wind during DEs (Aili et al., 2022; Liu et al., 2004; Tian et al., 2019). Microorganisms possessing the ability to transform and utilize these pollutants might have the advantage of thriving in polluted DEs. Our 16S rRNA analysis revealed pollutant‐degrading genera, including *Comamonas* (Lu et al., 2022), *Sphingomonas* (Zhou et al., 2022), *Comamonadaceae* (Fahy et al., 2006) and *Acinetobacter* (Tesso et al., 2019) in Proteobacteria, as well as *Hymenobacter* (Guo et al., 2020) and *Frankiales* (Wang et al., 2021) in Bacteroidota, and *Bacillus* (Ikram et al., 2022) in Firmicutes. These microorganisms have the potential to degrade a wide range of organic pollutants.

The genus *Staphylococcus* demonstrated consistent activity across all samples (Figure 2B) and its close interactions with other genera (Figure 2C) suggest its key role in the dust‐borne community. Different *Staphylococcus* species are known for their tolerance to high salt concentrations and arid conditions, enabling their survival and long‐distance travel during DEs (Feng et al., 2022; Kozajda et al., 2019; Tsai et al., 2011). Additionally, certain *Staphylococcus* species can induce quorum sensing (Lyon & Novick, 2004; Otto, 2009), promoting inter‐bacterial communication and collaboration through signaling molecules, supporting coexisting bacterial adaptation in unstable environments (Gobbetti et al., 2007; Novick & Geisinger, 2008). Moreover, *Staphylococcus* species (Hou et al., 2018), along with *Cutibacterium*, *Bacillus,* and *Paenibacillus* (Arnaouteli et al., 2021; Coenye et al., 2022; Timmusk et al., 2019), have been found to release extracellular polymeric substances, supporting biofilm formation, nutrient supply, and attachment in the community. Prolonged exposure to airborne dust particles can result in various health issues, including conjunctivitis, meningitis, and coccidioidomycosis (Aghababaeian et al., 2021). Nevertheless, the causing factors are not fully understood. While a range of potential pathogenic microorganisms have been identified during DEs, their viability remains unknown.

Of particular note is *Staphylococcus*, consistently detected in nearly all samples, suggesting its widespread presence during DEs. This species has been reported to be abundant (Kakikawa et al., 2008) and bioactive (White et al., 2020) in other DEs. Beyond its activity in the microenvironment, certain *Staphylococcus* species are associated with human pathogens (Balasubramanian et al., 2017; Vestergaard et al., 2019), that could induce dust‐associated health effects.

Another key genus in our dust samples is the spore‐forming *Bacillus*, which includes potential traits such as biomineralization (Keren‐Paz et al., 2022), biofilm formation (Ma et al., 2017), and toxicity (Azarkar & Zare Bidaki, 2016). Other studies have managed to detect and isolate *Bacillus* species in dust and other high‐altitude samples, and it is hypothesized that their survival is due to sporulation abilities (Griffin, 2004; Wainwright et al., 2003; Yoo et al., 2019). An interesting phenomenon is observed when considering the association between the abundance of spore‐forming microorganisms and both the total number of ASVs and the abundance of potentially active microbial ASVs. Notably, the ASV count for DE_III‐N stands out as the highest of all samples (431; Figure 1D), and the relative abundance of spore‐forming microorganisms, including *Bacillus*, *Paenibacillus* and *Lactobacillus*, also peaks in both total and potentially active ASVs, at 4.1% and 9.8%, respectively (Tables S1 and S2). In contrast, a very low relative abundance of spore‐forming microorganisms was observed in DE_II, consistent with a decrease in the total counts and potentially active ASVs (Figure 1C,D). Therefore, it is hypothesized that the presence of spore‐forming microorganisms positively affects the dust‐borne community composition and the active microorganism survival.

In conclusion, our results demonstrate positive interactions between potentially active dust‐borne communities, involving collaborative metabolic processes such as element cycling, pollutant degradation and biofilm formation. These interactions likely play a role in microorganism survival and adaptation in the challenging dust environment, while also potentially influencing broader phenomena like biogeochemical cycling and implications for human health. Further exploration could shed light on microbial resilience and adaptation in extreme environments and may pave the way for novel insights into the broader implications of these versatile microorganisms.

## AUTHOR CONTRIBUTIONS

**Naama Lang‐Yona:** Conceptualization (lead); resources (lead); supervision (supporting); writing – review and editing (lead). **Pengfei Hu:** Data curation (lead); formal analysis (lead); investigation (equal); methodology (equal); writing – original draft (lead). **Yehonatan Sharaby:** Formal analysis (supporting); methodology (equal); validation (equal); writing – review and editing (equal). **Ji‐Dong Gu:** Resources (equal); supervision (equal); writing – review and editing (equal). **Adi Radian:** Resources (equal); supervision (equal); writing – review and editing (equal).

## CONFLICT OF INTEREST STATEMENT

The authors declare no conflicts of interest.

## Supporting information


**DATA S1.** Supporting Information.

**TABLE S1.** Abundance and taxonomic ranking of amplified sequence variants (ASVs) of 16S rRNA gene amplified from DNA of the collected dust sample.
**TABLE S2.** Abundance and taxonomic ranking of amplified sequence variants (ASVs) of 16S rRNA amplified from cDNA of the collected dust sample.
**TABLE S3.** Correlation matrix of dust‐borne active bacteria at the genus level.
**TABLE S4.** Species distribution at different dust events, based on lowest taxonomical rank identified.

## Data Availability

The genomic sequencing data supporting the results have been archived in the NCBI GenBank Database under the accession number: PRJNA982604.
