# Supplementary material for: Environmental processes and health implications potentially mediated by dust‐borne bacteria
Source: Environ Microbiol Rep. 2023 Dec 27;16(1):e13222. doi: 10.1111/1758-2229.13222 (PMC10866058; doi:10.1111/1758-2229.13222)
Supplement: Supplementary file 1 — DATA S1. Supporting Information. [file EMI4-16-e13222-s002.docx]

**Supporting Materials and Methods**

**Environmental parameters and backward trajectory analyses.** Data on wind speed, direction, temperature, and relative humidity were obtained from the Israel Meteorological Service (https://ims.gov.il/en), and particle concentration for particulate matter <2.5 µm and 10 µm (PM 2.5 and PM10, respectively) were obtained from the Israeli Environmental Ministry database (https://air.sviva.gov.il/), both from nearby monitoring stations. The origin of sampled dust was determined by calculating back trajectories using the hybrid single-particle LaGrangian integrated trajectory model (HYSPLIT; https://www.ready.noaa.gov/HYSPLIT_traj.php). Each back trajectory map was computed for 48 hours at 200 meters above ground level, with trajectory obtained every 1 h for the duration of the sampling (*i.e.*, 6 trajectories for 6 h sample).

**DNA/RNA extraction, amplification, and sequencing.** The DNA/RNA extraction was performed using the ZymoBIOMICS DNA/RNA Kit (Zymo Research, California, US). The DNA extracts were stored at -80 °C, while RNA extracts were promptly utilized for complementary DNA (cDNA) synthesis. The remaining RNA was kept at -80 °C. For cDNA synthesis, Qiagen's QuantiTect Reverse Transcription Kit (Cat #: 205313, Hilden, Germany) was employed. The total cDNA reaction of 20 µL consisted of 1x reverse transcription (RT) enzyme, 4 µL of RT mix, 1 µL of random primer mix, and 5 µL of RNA extract. Thermocycling was performed using the MiniAmp Plus Thermocycler (Applied Biosystems, Thermo Fisher Scientific, Massachusetts, US). The thermocycling program included 25.0°C for 3 min, 45°C for 10 min, 85°C for 5 min, and 10°C until the process was terminated. The resulting cDNA samples were stored at -20°C for subsequent analysis.

The bacterial 16S rRNA gene and 16S rRNA were amplified using the primers CS1 515F - CS2 926R targeting the biodiversity and active community compositions (Walters *et al.* 2016). The amplification was performed in a 25 µL reaction volume containing a polymerase mix of 12.5 µL, 400 nM of each primer, 5 µL of DNA/cDNA template, and water. The cycling program consisted of an initial denaturation step at 95 ºC for 1 min, 30 cycles of 95ºC for 15 sec, 55ºC for 15 sec, 72ºC for 10 sec, and a final extension at 72ºC for 10 min. PCR products were verified on 1% agarose gel, and the triplicates were pooled. Gel visualization was performed using a ChemiDOC^TM^ MP Imaging System (BioRad, California, US).

**High-throughput sequencing and taxonomic analysis.** High-throughput sequencing was conducted at the Technion-Israel Institute of Technology, Haifa, using the Illumina Miniseq platform, with a sequencing depth of 100 kbp per sample. Sequence analysis was performed using QIIME2. Raw data from each sample were introduced into QIIME2 for quality control following the standard procedure (Bolyen *et al.* 2019). Specifically, raw sequences were denoised and filtered for invalid reads, mitochondrial sequences, and chloroplast sequences contamination using the software packages DADA2 (Callahan *et al.* 2016) and Deblur (Hanshew *et al.* 2013; Rowan-Nash *et al.* 2019). After quality control, taxonomic assignment of the amplicon sequence variants (ASVs) was performed using dada2 against the SILVA 138.1 reference database (Quast *et al.* 2012). Biodiversity was assessed using Chao1, Abundance-based Coverage Estimator (ACE), Shannon, and Simpson indices, as well as principal component analysis (PCoA) was calculated using the Vegan package after normalizing the sequence number of the 16S rRNA gene. Statistical significance between groups was calculated using Permutational multivariate analysis of variance (PERMANOVA). Venn diagram was contracted using the ggvenn package. The relative abundance values were calculated by averaging the ASVs of two sequenced sample replicates. The sequence data has been deposited in the NCBI GenBank Database under the accession number: PRJNA982604.

**Network construction of active microorganisms.** The interactions within the core dust microbial communities were investigated using a network of active microorganisms by calculating Spearman correlation coefficients between genera (Barberán *et al.* 2012; Varsadiya *et al.* 2021). Microorganisms from the top five phyla were merged at the genus level, as pre-determined from the 16S rRNA result. A two-by-two correlation matrix (Table S3) was generated using the “Hmisc” package in R (Varsadiya *et al.* 2021). The false discovery rate (FDR) controlling procedure was used to calculate *p*-values, and the Benjamini-Hochberg method was applied for correction (Benjamini & Hochberg 2018). Correlation coefficients with an absolute value of 0.6 or greater, or -0.6 or less are defined as the cut-off for a meaningful co-occurrence network, with *p*-values < 0.01. Network visualizations were generated using the “igraph” package in R (Csárdi & Nepusz 2006). In the undirected network, nodes represent genera and edges represent correlations between nodes.


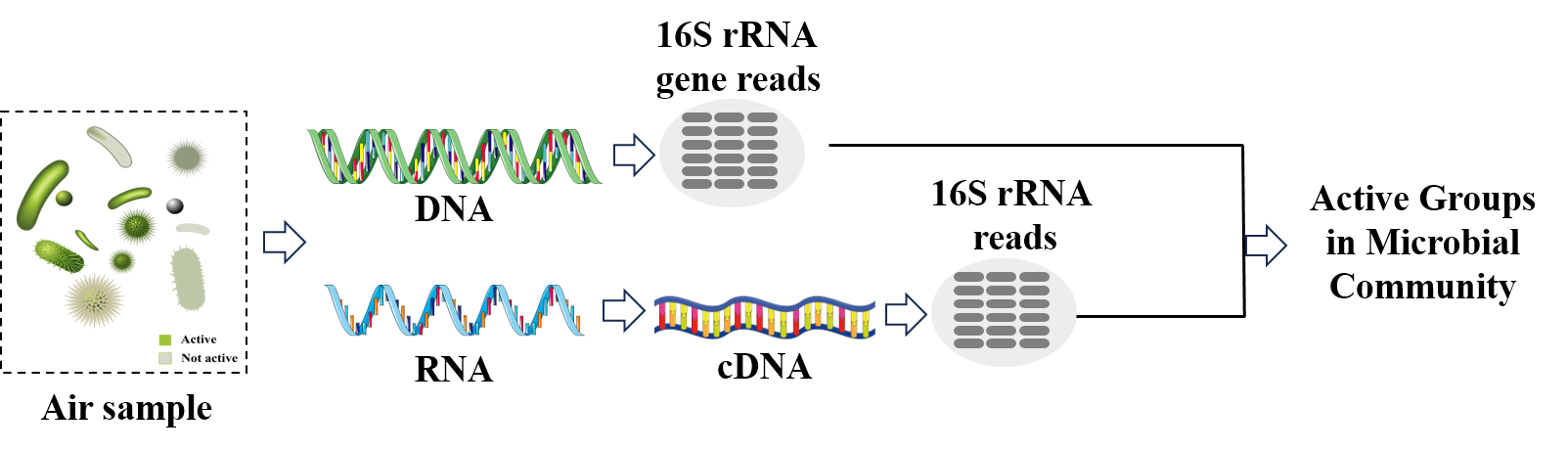


**Figure S1. Nucleic acid analysis workflow.** The simultaneous extraction of the DNA and RNA (converted to cDNA) was followed by amplicon sequencing of the 16S rRNA gene and 16S rRNA, respectively.


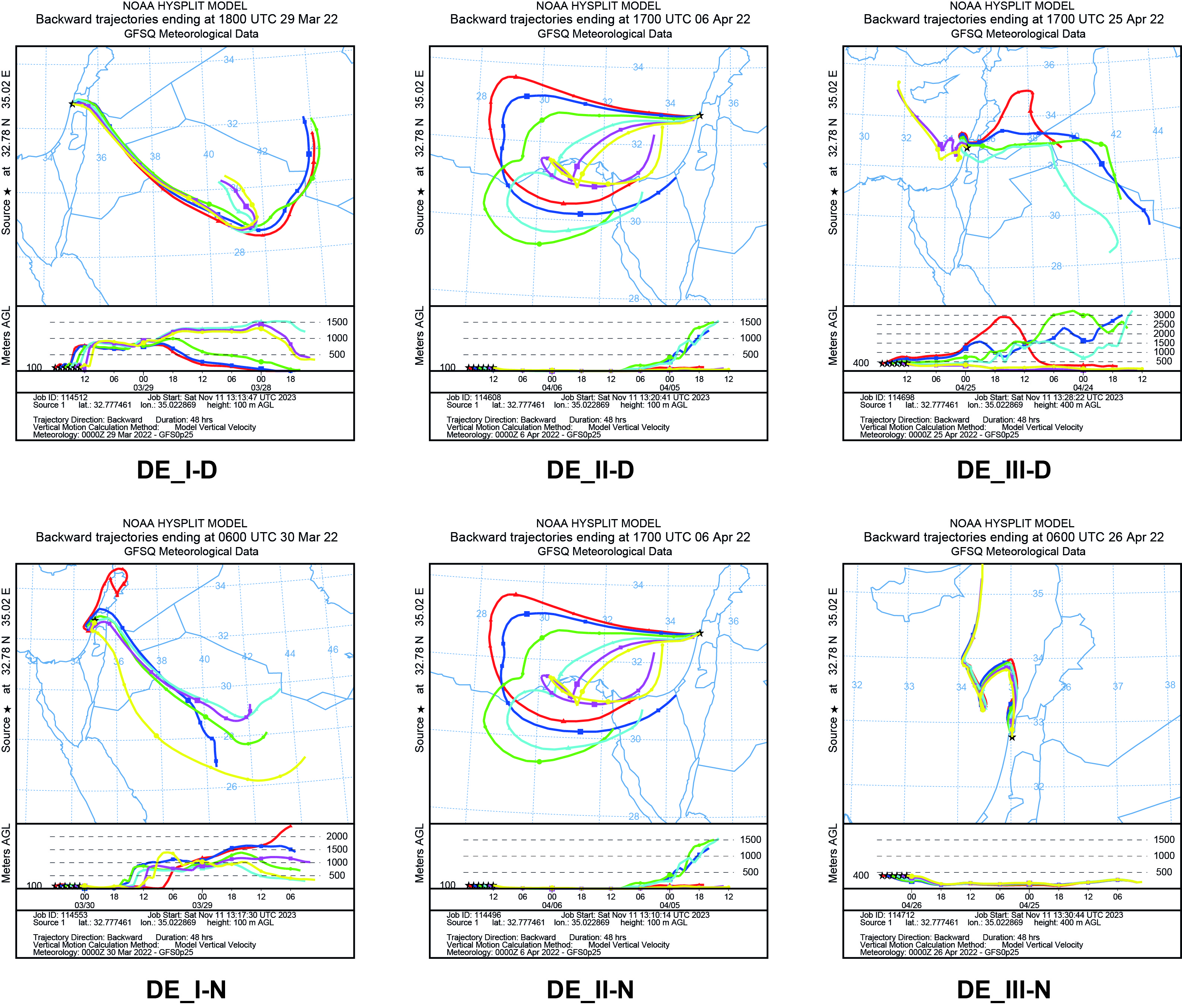
 **Figure S2.** **Airmass back trajectory analysis of the sampled dust events.** Forty-eight hours back trajectories analysis of air mass was calculated for the sampled dust events. Both day (_D) and night (_N) samples are analyzed.


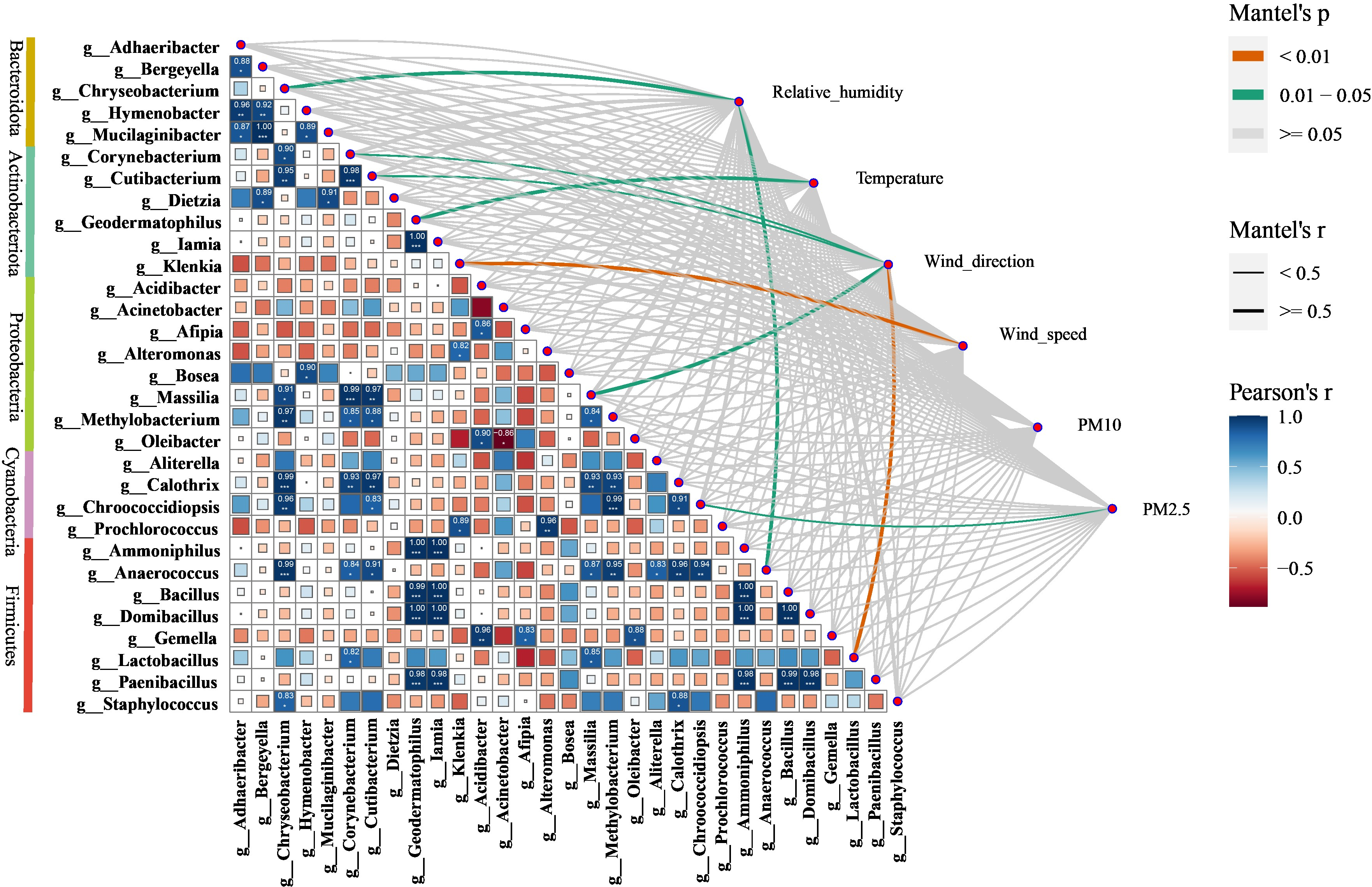


**Figure S3**. **Dust-borne microbial correlations with environmental parameters.** Correlation analyses were conducted to test correlations between potentially active species interconnections (Pearson) and environmental parameters (Mantel test).
